# Valproate-Induced Model of Autism in Adult Zebrafish: A Systematic Review

**DOI:** 10.3390/cells14020109

**Published:** 2025-01-13

**Authors:** Diletta Camussi, Maria Marchese, Ferdinando Nicoletti, Filippo Maria Santorelli, Asahi Ogi

**Affiliations:** 1 IRCCS Stella Maris Foundation, 56128 Pisa, Italy; 2 Department of Physiology and Pharmacology Vittorio Erspamer, “La Sapienza” University of Rome, 00185 Rome, Italy; 3 IRCCS Neuromed, 86077 Isernia, Italy

**Keywords:** adult zebrafish, social behavior, autism spectrum disorder, valproic acid, valproate

## Abstract

Autism spectrum disorder (ASD) is a neurodevelopmental disorder characterized by deficits in social skills and the presence of repetitive and restricted behaviors and interests. The social behavior of the zebrafish (Danio rerio) makes this organism a valuable tool for modeling ASD in order to explore the social impairment typical of this disorder. In addition to transgenic models, exposure of zebrafish embryos to valproic acid (VPA) has been found to produce ASD-like symptoms. This review first sets out to examine the existing literature on adult social behavior in the zebrafish VPA-induced model of autism, and the authors also aim to identify the ideal VPA dosage able to induce a persistent and long-lasting ASD-like phenotype while minimizing the suffering and distress of research animals in compliance with the principles of replacement, refinement, and reduction (3Rs).

## 1. Introduction

Valproic acid (VPA) is a branched short-chain fatty acid used in humans as an antiepileptic drug (AED) and as a mood stabilizer in many neurological and psychiatric diseases, such as epilepsy, migraine, and bipolar disorder [1,2]. Its mechanisms of action, not yet fully elucidated, involve different pathways: VPA inhibits gamma-aminobutyric acid (GABA) transaminase and increases the synthesis of GABA by enhancing the expression and activity of glutamic acid decarboxylase, these actions leading to increased GABA levels in the brain; VPA also modulates the conductance properties of the sodium, potassium, and calcium channels [3]; finally, it exerts an epigenetic function, inhibiting histone deacetylase (HDAC), specifically HDAC1 [4].

To further elucidate the mechanisms of action of VPA and its effects in different clinical conditions, numerous and diverse animal models have been developed [5,6,7,8,9]. In recent years, the zebrafish has emerged as a particularly valuable experimental model since its developmental stages, and responses to drug exposure are easily studied in vivo [10]. Indeed, because of its high genetic and physiological homology to mammals, as well as certain intrinsic features, namely its high fecundity, external fertilization and development, optical transparency through the early larval stages (this latter feature making its central nervous system (CNS) readily accessible), and short lifespan, it lends itself to experimental manipulation (genetic and otherwise) and systematic drug screening and discovery [11,12,13,14,15]. Zebrafish models of autism spectrum disorder (ASD) and other conditions have been developed for two purposes: to assess the protective and beneficial effects of VPA in these different conditions and to explore VPA-induced toxicity at morphological, behavioral, and molecular levels, i.e., both within and outside the CNS [16,17,18].

### 1.1. Beneficial Effects of VPA

The zebrafish has been shown to be suitable for studying epilepsy pathophysiology and treatment in vivo. The epileptic phenotype is classically induced by a variety of chemoconvulsants, primarily pentylenetetrazol (PTZ), kainic acid, and pilocarpine [19]. The PTZ-induced zebrafish model of epilepsy has been used to investigate the effects of established AEDs, such as carbamazepine, gabapentin, diazepam, and VPA [20]. The latter has been shown to suppress seizure-like behavior, such as excessive involuntary activity, and improve learning abilities in PTZ-treated adult zebrafish [21]. Indeed, VPA-pretreated zebrafish with PTZ-induced seizure-like behavior displayed a lower seizure intensity and a significantly delayed onset of seizures compared with what was observed in non-pretreated animals; furthermore, learning ability, impaired by PTZ, also improved in this model [21].

Notably, the behavioral and antiepileptic effect of VPA has also been confirmed at the molecular level by reduced mRNA expression of heat shock protein 70 kD [22], c-fos (a marker of neuronal activity) [23], and cAMP (a transcriptional regulator) [24]. Attenuation of PTZ-induced epileptic manifestations with VPA provides a reference zebrafish model for the screening of antiepileptogenic agents [24]. Elegant reviews provide a more comprehensive overview of epilepsy modeling in zebrafish [25,26]. Notably, Baraban et al. [27] reported that *scn1labs552*-mutant zebrafish replicated many features of Dravet syndrome (DS), a genetic epilepsy of childhood [28]. Screening of AED efficacy showed VPA to be a significant suppressor of electrical seizure activity in the brain and an effective agent in behavioral seizure assays [27,29]. Beyond the PTZ-induced and DS models, several other models of genetically determined epilepsies, such as Lafora disease [30], have been developed [16].

Moreover, recent research in animal models of other neurological conditions, such as spinocerebellar ataxia-3, radiation-induced neuronal injury, and stroke, demonstrated a neuroprotective effect of VPA, which appears to be dependent on HDAC-mediated pathways [4,31,32,33,34]. VPA is FDA-approved for the treatment of a range of neurological and psychiatric conditions, such as seizures, in both adults and children, and it has also been shown to be useful in manic episodes in bipolar disorder, as well as in migraine and postherpetic neuralgia [35].

### 1.2. Toxic Effects of VPA

Most studies investigating the therapeutic effects of VPA also described adverse events related to VPA exposure [33], often displaying a time- and dose-dependent pattern. Hepatotoxicity in zebrafish after embryo VPA exposure is explored through assessment of common hepatotoxicity-related markers, which have shown upregulation of pathways associated with oxidative stress and xenobiotic metabolism and downregulation of processes involved in ribosome and protein kinase activity and regulation of transcription [36]; nephrotoxicity and reproductive toxicity have been extensively investigated and found to be related to increased reactive oxygen species production and mitochondrial dysfunction [33].

Moreover, VPA is considered to be a potent teratogenic compound linked to diverse effects in humans, especially when consumed by pregnant women during the first trimester of pregnancy [37]. Indeed, prenatal exposure to VPA has been linked to an increased risk of developing three possible spectra of alterations: major congenital malformations (such as heart defects, cleft palate, abnormalities of the urinary tract and limbs, spina bifida), valproate syndrome (characterized by facial dysmorphism and neural tube defects), and neurodevelopmental disorders, such as ASD [38,39]. According to the American Psychiatric Association’s Diagnostic and Statistical Manual of Mental Disorders (DSM-5), ASD is a neurodevelopmental disorder characterized by impaired social communication, repetitive behaviors, and cognitive deficits [40]. Due to its intrinsic features and its neurodevelopmental homology to mammals, the zebrafish has been widely validated as a viable model for analyzing the neurodevelopmental causes of autism [41]. In particular, the VPA-induced zebrafish model of autism, obtained through the administration of VPA during critical neurodevelopmental windows [42], has made it possible to shed light on the mechanisms underlying ASD pathophysiology [43].

Numerous zebrafish studies exploring drug-induced neurodevelopmental disorders have focused on the disruption of morphological features, early behavioral characteristics, and molecular profile changes [42,44]. These aspects have been extensively explored in zebrafish embryos up to 7 days post-fertilization (dpf). Legradi et al. [45] provided a comprehensive literature review of studies conducted within 7 dpf. We have observed that most of the behavioral research on larvae was conducted within this timeframe, but there is no clearcut development-related reason for this choice. To date, however, no systematic reviews of the literature regarding VPA-induced models of disease in zebrafish older than 7 dpf were performed. Consequently, to avoid overlapping with the existing review and to propose a new literature dissertation, we decided to focus the present manuscript on zebrafish older than 7 dpf.

Based on Directive 2010/63/EU on the protection of animals used for scientific purposes, zebrafish is typically considered “independently feeding larval form” and subject to regulations starting from 5 dpf [46]. This is a crucial developmental stage characterized by the initial acquisition of a functional digestive tract, the ability to move through the water column, and hunting for prey [46]. These characteristics consolidate between stages 5 and 7 dpf [47], as demonstrated by the full yolk absorption, which occurs at 7 dpf [48]. Moreover, a wide range of imaging techniques is effective for zebrafish embryos and larvae up to 7 dpf because, at this age, zebrafish lose their translucency [49]. At 7 dpf, dermal diffusion is still capable of meeting the O2 requirements, and blood circulation is still not essential, but gills begin to be needed for ionoregulation [50].

Zebrafish larvae develop key neural circuits and behaviors by 7 dpf, making it an ideal time point to start studying neurobehavioral effects [51,52]. For example, there is evidence suggesting that social preference may begin to develop as early as one week for the refinement of vision and coordination [53,54].

Comparative timelines of zebrafish and mammals are not easy to define because individual organs or tissues have different development timelines and, in some cases, because of the presence or absence of specific organs or tissues among different species. For example, there are comparative studies on zebrafish and mouse hematopoiesis timelines [55]. Noteworthily, it is known that the typical age-related phenotypes of zebrafish, such as lifespan and brain clearance systems, display similarities to humans but not to mice [56]. However, in general, 7 dpf is considered the stage equivalent to the postnatal period of mammalian species [57].

Finally, to obtain the ASD-like phenotype, the literature proposes different VPA dosages administered at different time points. In short, the models described are diverse, and there is no uniform consensus. For this reason, the aim of the present manuscript is to identify, through a systematic review of all available studies conducted on zebrafish older than 7 dpf, the ideal ASD model-shaping dose of VPA.

## 2. Materials and Methods

A literature search of the PubMed and Scopus databases up to 23 April, 2024 was performed using the terms “zebrafish” AND (“valproate” OR “valproic acid”). The search yielded 396 matches, but 144 articles were removed because they were duplicate records; a further 3 articles were removed because they were not in English, and another 1 because the paper was not accessible. The remaining records (*n* = 248) were further screened, and 227 articles were excluded for the following reasons: 33 focused on extra-CNS effects of VPA; 37 did not deal with VPA-induced ASD-like symptoms and/or zebrafish; 2 were not original research papers; 77 evaluated VPA as an AED; 78 evaluated VPA effects within 7 dpf. In order to reduce the risk of bias, the screening process was performed independently by two reviewers, who read the 248 articles in completeness, defined the exclusion criteria, and selected the articles included in the present manuscript.

In particular, we did not impose temporal limitations on the VPA administration timing. However, only studies that conducted behavioral and molecular tests after 7 days post-fertilization (dpf) were included in the review.

This literature revision was written in accordance with Preferred Reporting Items for Systematic Reviews and Meta-Analysis (PRISMA) criteria [58]; the protocol was not registered.

## 3. Results

**Figure 1:**
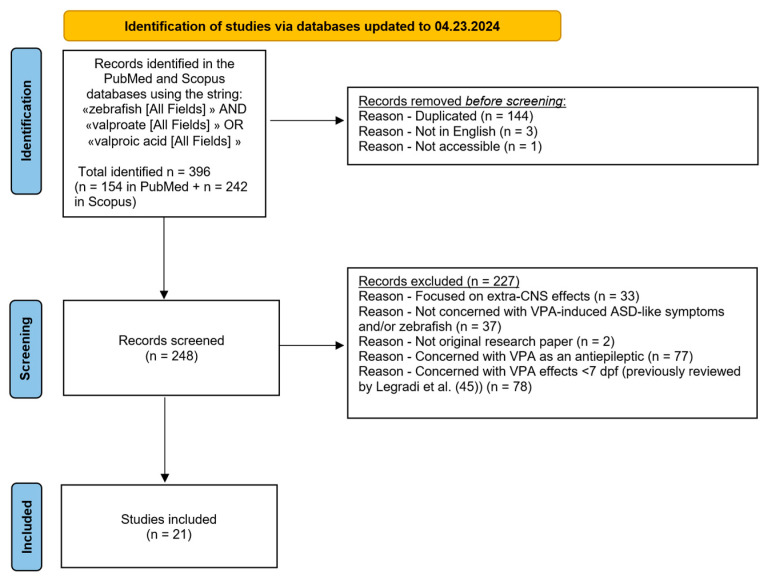
PRISMA flow diagram of the literature search process.

Overall, 21 articles were included in this review (see Figure 1 for a summary of the selection process).

They were then grouped into three categories: eight studies were carried out on VPA-exposed wild-type (WT) zebrafish larvae submitted to behavioral analysis in the juvenile stage of life (between 7 dpf and 90 dpf); nine were carried out on VPA-exposed WT zebrafish tested during the adult stage (older than 90 dpf) [59]; and four were conducted in both stages (see Table 1 for detailed data). Eighteen studies performed at least one behavioral test and sixteen at least one social behavior analysis. This review indeed showed that in adult zebrafish, behavioral impairment is the main expression of the ASD-like phenotype. When seeking to identify the most suitable VPA dosage to induce a persistent ASD-like phenotype (the main aim of the present review), we found the literature to contain contrasting findings, particularly in terms of non-social behaviors, which moreover were evaluated using a variety of tests and endpoints. As a result, we shifted our focus to social behavior, which was evaluated with greater consistency across the different studies and allowed better characterization of the zebrafish ASD model.

**Figure 2:**
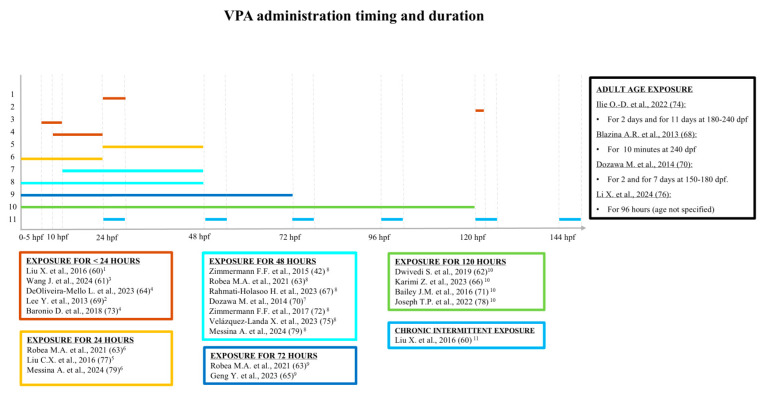
Timing and duration of VPA administration of the 21 studies included, hpf = hours post-fertilization. [42]^8^; [60]^1,11^; [61]^3^; [62]^10^; [63]^6,8,9^; [64]^4^; [65]^9^; [66]^10^; [67]^8^; [68] ^-^; [69]^2^; [70]^7, -^; [71]^10^; [72]^8^; [73]^4^; [74] ^-^; [75]^8^; [76] ^-^; [77]^5^; [78]^10^; [79]^6,8^.

**Figure 3:**
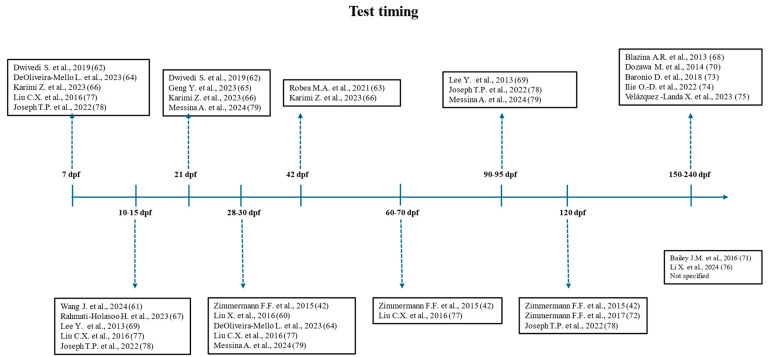
Timing of behavioral and molecular tests performed of the 21 studies included, starting from 7 dpf. Dpf = days post-fertilization [42,60,61,62,63,64,65,66,67,68,69,70,71,72,73,74,75,76,77,78,79].

To facilitate reading this review, the timing and duration of VPA administration are reported in hours post-fertilization (hpf) (Figure 2) and the age of the zebrafish, when tested, in dpf (Figure 3). Moreover, compared with the descriptions in the original reports, some behavioral analyses are here renamed and differently grouped, a “liberty” taken by the present authors in view of the absence of uniformity in the definition of behavioral tests. For example, “open field” and “novel tank” are generic terms commonly used to define similar, if not identical, tests with the same endpoint. Therefore, locomotor activity was divided into “motor coordination” (when assessing purely swimming parameters) and “hyperactivity”, “thigmotaxis”, or “bottom dwelling” (when assessing exploration also as a function of anxiety). Analysis of the light/dark (L/D) condition was divided into “L/D response” (behavioral response to sudden changes in brightness), “L/D activity” (circadian rhythm aligned sleep), and “shoaling L/D background exploration” (scototaxis). The term “social interaction” is here taken to mean “social preference” [80]; “social contact”, on the other hand, is a different test even though it has the same endpoint.

The main findings of the review are listed chronologically, from the least recent to the most recent.

### 3.1. Studies Analyzing Juvenile Specimens (Younger than 90 dpf)

- Liu et al. [60] exposed 24 hpf zebrafish larvae to either chronic intermittent treatment (VPA 20 µM for 7 h/day for 6 days) or acute treatment (VPA 100 µM for 7 h). At 30 dpf, they observed a deficit in locomotion only in the acutely treated zebrafish and a social behavior impairment only in the chronically exposed zebrafish. No difference between the two groups was observed in thigmotaxis or responses to sudden changes in light, which means that no anxiety-like behavior was observed.
- Wang et al. [43,61] exposed larvae to increasing concentrations of VPA (5–500 µM) for 8–120 hpf and observed ASD-like behavior at 10–13 dpf, even with just 5 µM VPA. Impairment of social behavior was assessed by evaluating shoaling, shoaling L/D background exploration, social contact, and the mirror biting test.
- Dwivedi et al. [62] exposed larvae to 75 µM VPA for 4–120 hpf. At 7 dpf, molecular (qRT-PCR and Western blot), behavioral (social preference and thigmotaxis, inattentive behavior), and non-behavioral (circling) tests were performed. The social preference test, as previously performed by Dreosti et al. [62], was conducted at 21 dpf, and the authors observed a severe social impairment in the treated zebrafish. They also administered drugs approved for the treatment of ASD-related symptoms (including aripiprazole and risperidone) and achieved their goal of reducing the abnormal behaviors and further confirming the phenotype.
- Robea et al. [63] exposed zebrafish larvae to 48 µM VPA for 24, 48, or 72 hpf. They studied the impact of early administration of VPA on zebrafish sleep patterns and on autistic-like social phenotype through social preference and the mirror biting test. Exposure to 48 µM VPA, even just for 24 h, was found to be sufficient to induce a significant social impairment persisting until at least 42 dpf. Instead, no difference in sleep pattern was observed at 6 dpf.
- DeOliveira-Mello et al. [64] explored the relationship between VPA-induced ASD-like behavioral alterations and visual impairment by exposing Turku strain zebrafish to 25 µM VPA for periods ranging from 10 to 24 hpf. They performed immunostaining and gene analysis and observed a delay in the early development of the retina and optic nerve at ≃7 dpf, while no significant difference in visual function, assessed through optomotor behavior and color preference test, was detected at 30 dpf. In contrast with Robea et al. [63], they observed abnormal sleep-like behavior at 7 dpf.
- Geng et al. [65] exposed zebrafish larvae to 1 µM VPA for up to 72 hpf, and then, at 21 dpf, they treated some zebrafish for 1–3 h with 237 neuroactive compounds, known to interfere with social behavior. Right after the treatment, they performed the “ZeChat”, a social contact assay exploring unsupervised deep learning, to characterize sociality. They observed a significant social impairment in the group treated with VPA alone and increased sociality in the zebrafish exposed to VPA and D3 receptor agonists.
- Karimi et al. [66] exposed zebrafish larvae to VPA concentrations of 1–75 µM for up to 120 hpf. Taking into account survival rate and teratogenic side effects, they chose a concentration of 1 µM to perform molecular Wingless/Integrated (WNT)-related pathway analysis at 7 dpf. Also, an inattentive behavior test was performed at 7 dpf, which highlighted a lack of response to aversive stimuli in treated zebrafish embryos compared with controls, which could be taken as a sign of learning impairment. The social preference test was performed at 21 dpf, revealing a social interaction impairment in the treated zebrafish. To investigate the long-lasting impact of 1 µM VPA, they conducted further tests at 42 dpf without, however, observing significant differences between the exposed fish and the controls.
- Rahmati-Holasoo et al. [67] treated zebrafish larvae with 48 µM VPA for up to 48 h and, similarly to Wang et al. [61], evaluated shoaling, social contact, mirror biting, and shoaling L/D background exploration at 10–13 dpf. They also administered oxytocin, a nonapeptide known to be crucial in modulating social behavior [81]. Interestingly, despite not using isotocin, the bony fish analog of mammalian oxytocin [82], they observed a recovery of social and aggressive behavior.

### 3.2. Studies Analyzing Adult Specimens (90 dpf and Older)

- Blazina et al. [68], exposing 240 dpf adult zebrafish to high doses of VPA (88, 265, 884 µM) for 10 min, found impaired swimming behavior, used as a measure of social behavior, at these high concentrations.
- Lee et al. [69] explored the link between altered cell proliferation in telencephalic regions and ASD-like phenotype in adult fish. They acutely exposed fish embryos to high-dose (2000 µM) VPA for 3 h at 5 dpf. Despite confirming the molecular phenotype through qRT-PCR at 95 dpf, they found no difference in passive avoidance learning or on the bottom dwelling anxiety test. Their findings suggest that the VPA effect is time- and timing-dependent, regardless of the administration of a high dose of the drug.
- Dozawa et al. [70] explored the effect of VPA as an HDAC inhibitor. They exposed adult zebrafish to 500 µM VPA (for 48 or 168 h, depending on the outcome being sought) and performed a cell linage analysis, immunostaining, and in situ hybridization, confirming findings previously reported in mouse models [83]. VPA inhibits HDAC activity and upregulates Notch signaling, which in turn reduces cell proliferation in the optic tectum of adult zebrafish. The HDAC–Notch pathway is critical for proper neurodevelopment [84] and is known to be altered in multiple neurological and psychiatric disorders [85]. Given VPA’s inhibitory function on HDAC, it could be hypothesized that a disruption (particularly an early disruption) of the precise spatial and temporal regulation of this pathway could be linked to the development of molecular and behavioral ASD-associated traits, particularly social behavior-related ones, in both humans and zebrafish.
- Bailey et al. [71] exposed zebrafish larvae to increasing concentrations of VPA (0.5, 5, or 50 µM) for ≃120 hpf. They evaluated adult zebrafish (age not specified) using a neurobehavioral test battery (including startle reflex habituation, novel tank, shoaling behavior, and predator avoidance). The VPA-treated fish showed no significant difference in startle reflex habituation or predator avoidance but, in contrast, displayed hyperactivity in the novel tank exploration assay. Moreover, even just 5 µM VPA negatively affected shoaling behavior. Based on their observations, the authors concluded that possible motor or visual impairments were not likely to be the cause of the observed behavioral differences. These data are consistent with results obtained in juvenile animals by DeOliveira-Mello et al., who, using 25 µM VPA, did not find long-lasting retinal impairment [64].
- Zimmermann et al. [72] conducted qRT-PCR, high-performance liquid chromatography (HPLC), and enzymatic activity assays on their previously characterized zebrafish model of autism [42]. They found a purinergic system dysfunction at 120 dpf, confirming their previous findings, and proposed a possible pathway underlying social behavior impairment.
- Baronio et al. [73] performed qRT-PCR and HPLC to investigate VPA-induced alterations of the histaminergic system in both larvae and adult zebrafish. They exposed Turku strain zebrafish larvae to 25 µM VPA from 10 to 24 hpf and performed, at 180 dpf, hyperactivity and social preference tests. No significant difference was found in locomotion between the treated and control groups. On the contrary, a significant link between social impairment and histaminergic system disruption was detected, supporting the hypothesis that the histaminergic system contributes to the development of ASD-like symptoms [86].
- Ilie et al. [74] used 18 µM VPA for both acute (2-day-long) and chronic (11-day-long) treatment in adult zebrafish (180–240 dpf). Immediately after the treatment, they performed social preference and mirror biting tests, finding disrupted social behavior and increased aggressivity in both the acutely and chronically treated fish.
- Velázquez-Landa et al. [75], on the basis of human evidence regarding sexual behavior-related problems in social interaction in ASD [87], endeavored to “contribute to the knowledge related to sexual behavior in this disorder” using a zebrafish model of autism. They adopted a previously described protocol [42], exposing zebrafish larvae to 48 µM VPA for 48 hpf and, at 180 dpf, observed a disruption of sexual behavioral stages in VPA-treated fish, with the females displaying reduced oviposition.
- Li et al. [76], like Ilie and colleagues [74], did not perform early-stage VPA administration but directly exposed adult fish (age not specified) to a high dose (500 µM) of VPA for 4 days. They performed social preference and mirror biting tests, finding social impairment but, unlike Ilie et al., a decrease in aggression. Moreover, they found anxiety-like behavior, assessed through a bottom dwelling test and a hyperactivity test.

### 3.3. Studies Analyzing Both Juvenile and Adult Specimens

- The first study in both juvenile and adult zebrafish was conducted by Zimmerman and her group [42], who demonstrated effects on ASD core symptoms of early VPA exposure. They exposed zebrafish larvae to 48 µM VPA for 48 hpf and then performed analyses of both anxiety-related behavior (hyperactivity and bottom dwelling tests at 6, 30, 70, and 120 dpf) and social behavior (social preference and mirror biting tests at 70 and 120 dpf). They found hyperactivity at 6 dpf but not at 30, 70, or 120 dpf. They also found an increase in anxiety up to 70 dpf, while an anxiolytic effect was observed at 120 dpf. A social behavior impairment was detected at both 70 and 120 dpf, while no difference in aggressive behavior was detected.
- Liu et al. [77] focused on the characterization of the expression of *shank3,* a gene strongly implicated in the pathogenesis of ASD and related syndromes [88]. These recent findings demonstrated a critical role for *shank3* in synaptic function and in social interaction and communication, providing a functional link between *shank3* and ASD behavioral features. To assess the viability of a zebrafish model of autism for elucidating *shank3* involvement in the etiopathogenesis of ASD, Liu et al. exposed zebrafish embryos to 500 or 1000 µM VPA at 24–48 hpf. They observed altered differential expression of *shank3* isoforms at different time points (1 to 60 dpf) [77].
- Joseph et al. [78] utilized a VPA-induced ASD zebrafish model to verify the protective function of duloxetine (DLX, a serotonin and noradrenaline reuptake inhibitor) on hyperactivity, anxiety-like behavior, and social deficit. They formed two groups of specimens aged from 8 hpf to 108 hpf; one group was treated with 10 µM VPA, and the other also with increasing doses of DLX. After the treatment, they performed an L/D response and a social contact assay in juvenile fish (5–7/13 dpf). They then performed social preference, hyperactivity, and bottom dwelling tests in adult fish (90–120 dpf). Hyperactivity, increased anxiety-like behavior, and social deficits were found to persist up to 120 dpf, while 20 µM DLX was found to rescue ASD-like features.
- Messina et al. [79] focused on the potential link between loss of brain asymmetry and social cognition impairment on the basis of the consideration that the right hemisphere seems to be mainly linked to emotional and social processing and the left one to attention and categorization [89,90]. A lack of left visual bias (i.e., of the predominant use of the left eye) has been shown to be related to impaired facial expression recognition [91] and seems to characterize some neuropsychiatric disorders, such as ASD [92]. Building on their previous study [93], the authors exposed zebrafish larvae to 1 µM VPA for 24–48 h and then performed the visual mirror test (21 dpf) to assess possible reduction of left visual bias, a social preference test (28 dpf), and a molecular test to explore asymmetric lateralization gene expression (90 dpf). Both exposed groups exhibited decreased social preference and a reduction of left visual bias. Moreover, the molecular analysis (qRT-PCR) showed a neutralization of the asymmetric distribution of genes responsible for brain lateralization [79].

## 4. Discussion

Zebrafish have been shown to be a particularly valuable model for translational behavioral neuroscience research, specifically for studying human social disorders, such as ASD [94,95], a neurodevelopmental disorder characterized primarily by impairment of social communication and interaction and by restrictive and repetitive patterns of behavior or activities [40]. This latter category also includes difficulties with transitions, inflexible thinking, and increased distress over minor changes, potentially leading to comorbidities, such as anxiety in new situations, or maladaptive behaviors, such as aggressive/self-harming behaviors and irritability [96].

The above-outlined symptomatology of ASD seems to be underpinned by monoaminergic system alteration, found in both human and animal models [97]. Disrupted modulation of this system may partially explain the social and communicative deficits typical of ASD [97]. Most of the FDA-approved drugs for ASD-related symptoms, such as aripiprazole and risperidone, target monoamines, namely dopamine (DA) and serotonin (5HT), and were previously shown in experimental studies to reduce both behavioral and structural abnormalities in VPA-induced ASD-like mouse models [98]. Along the same lines, Dwivedi et al. demonstrated the efficacy of these drugs in reducing behavioral symptoms in a VPA-induced zebrafish model of autism [62]. Maintenance of physiological 5HT levels is essential to preserve the excitatory/inhibitory balance in cortical neurons, and different studies in both human and animal models have consistently shown an impairment of serotoninergic neurotransmission [99]. With regard to behavioral phenotypes, numerous studies on rodent models of ASD found abnormal social behaviors, such as defective social interaction, repetitive behaviors, and increased anxiety, along with serotonergic system abnormalities [100,101]. Moreover, zebrafish models of autism display abnormal differentiation of serotoninergic neurons [102] and a brain-region-dependent quantitative reduction in these neurons, leading to circadian rhythm disruption, as discussed by DeOliveira-Mello et al. [64].

The brain’s catecholaminergic system, which includes DA and norepinephrine (NE), is known to be involved in the development of neurodevelopmental and neurological diseases [97]. Catecholaminergic abnormalities have been detected in VPA-treated animals and have been associated with behavioral and mood impairment, such as reduced social interaction, increased repetitive behavior, anxious and depressive moods, and impaired cognitive flexibility [103,104]. The dopaminergic pathway, in particular, seems to be strongly implicated in ASD pathophysiology, given its essential role in reward circuit modulation and, consequently, in social motivation and interaction [105,106]. Dopaminergic system alterations have been reported in studies of rodent models of VPA-induced ASD, with DA levels found to be increased or reduced in different brain regions, possibly as a result of differential expression of DA-related signaling molecules across the brain [107,108]. Consistent with findings in rodents, Geng et al. reported that administration of dopamine D3 agonists, as opposed to other DA receptor agonists, led to a significant reduction in social impairment in zebrafish with VPA-induced ASD-like traits [65].

Numerous studies have reported abnormalities in resting-state functional connectivity within the locus coeruleus, an NE-enriched brain region, in children with ASD [109]. Like dopaminergic alterations, dysregulation of NE levels appears to be highly dependent on the brain regions considered [110,111]. Increases in NE transporter expression and acetylation levels have been reported in VPA-treated rats [111]. Additionally, antipsychotic medications acting on NE receptors have been found to alleviate ASD symptoms [112]. In line with these findings, Joseph et al. observed a significant improvement in anxiety-like behavior, social interaction, and hyperactivity in zebrafish models of ASD following treatment with DLX [78].

Finally, the histaminergic system plays an important role in the modulation of the sleep–wake cycle, reward-seeking behaviors, neuroinflammation, emotion, learning, and memory [113]. Neuronal expression of various histamine (HI) receptors plays a key role in regulating neurological functions, such as neurogenesis, axogenesis, and cell type-specific differentiation [114]. Studies on postmortem human brains have shown elevated levels of enzymes and HI receptors in the dorsolateral prefrontal cortex of individuals with ASD, indicating a putative role for the histaminergic system in the pathophysiology of ASD [115]. In line with these findings, Baronio et al. found a significant correlation between social impairment and histaminergic system changes in VPA-exposed zebrafish [73].

As indicated in Table 2, we found no uniformity of behavioral analysis in terms of either tests performed or the definition of such tests. However, the tests used specifically to evaluate social behavior in VPA-exposed zebrafish can be summarized and grouped as follows: tests of social preference (used in *n* = 13 studies) and social contact (*n* = 4); evaluation of shoaling (*n* = 3), including one study which also considered shoaling predator avoidance, and shoaling L/D background exploration (*n* = 3); mirror biting test (*n* = 6); and, in one study, a visual mirror test. Social preference is generally evaluated by studying the avoidance/attraction response of a single fish to a social stimulus, generally a shoal [95], with social attraction (prosociality) taken as the outcome. Conversely, social contact (sometimes referred to as interaction) is usually tested by evaluating the orientation of and distance between two subjects in a one-to-one interaction [116]. For this reason, in the present review, “social interaction” tests that were used to assess social attraction are considered among the “social preference” ones. In addition, some one-to-one conditions could elicit possible agonistic behavior and potentially be used to measure aggressiveness, essentially becoming mirror biting tests. The shoaling test assesses the tendency of fish to aggregate in shoals, quantifying their spatial behavior and sociality [117], while the shoaling predator avoidance test involves exposing shoals to the image of a predator and measuring shoaling cohesion [118]. However, it should be highlighted that predator escape is mostly used to assess the fear (or anxiety) response rather than social behavior [119]. Mirror biting is a parameter used to measure aggression shown by a solitary zebrafish when confronted with its own mirror image; the test involves counting the number of times it attacks its image [120]. Differently, the visual mirror test is commonly used to assess left visual bias, the typical lateralized response to social stimuli [79].

As for other (non-social) behavioral tests, the aspect most commonly analyzed (in *n* = 9 studies) was locomotor activity. Locomotor activity analysis is often used to evaluate possible anxiety-like behaviors in zebrafish, even though humans affected by anxiety disorders can actually display either hypo- or hyperactivity [121]. It is well known, too, that freezing/immobility can constitute a stress response or anxiety-like behavior in zebrafish [122] and also in other species [123]. Movement patterns (locomotion) do not always reflect exploration (investigation of the environment); equally, they are not a mechanical expression of anxiety. For this reason, it may be inappropriate to indiscriminately associate hyperactivity with anxiety. Non-social behavioral analyses should seek to analyze exploratory behavior. In particular, the results of tests of thigmotaxis, bottom dwelling, or L/D exploration would be more appropriate indicators of an anxiety-like pattern. Moreover, anxious people are, in general, more prone to show inappropriate coping strategies [124], and individuals with ASD often struggle to manage and adapt to changes; clearly, then, the novelty of the stimuli presented (either environmental or social) could represent an extremely relevant aspect when testing zebrafish behavioral responses.

This review revealed a high heterogeneity of protocols used to induce ASD-like phenotypes in zebrafish. The VPA dosages used ranged from very low (less than 1 µM) to very high (exceeding 1000 µM), and the duration of exposure also differed greatly across studies, with some researchers treating zebrafish larvae acutely and others opting for chronic exposure (Table 1). Two research groups induced the ASD-like phenotype by directly exposing adult specimens to VPA [74,76].

With regard to ASD-like social behavior, data from the reviewed literature showed that early exposure to very low dosages of VPA (1–5 µM), regardless of the timing of exposure, can induce early social impairment detectable at 10–13 dpf [61], 21 dpf [65,66], and 28 dpf [71,79], but with no significant difference versus controls at 42 dpf [66]. Furthermore, a brief early administration of 5 µM VPA (8 to 12 hpf) produced social impairment at 13 dpf [61], while the same dosage used for 120 hpf led to social impairment at 60–90 dpf [71]. The same behavioral alterations, detectable at 30 dpf and beyond, were induced by exposing zebrafish to 10–25 µM, not only for 120 hpf [78] but also for just 14 hpf [64,73]. Conversely, at higher dosages (25 to 48 µM), VPA seems to be capable of inducing early and more persistent social impairment, lasting until at least 120 dpf after an exposure time of even just 24–48 h [42,63,64,67,78].

Conversely, chronic intermittent exposure (20 µM VPA for 7 h/day for 6 days) resulted in an impairment of social behavior at 30 dpf [60]. Considering that continuous VPA administration has comparable effects, the chronic intermittent protocol does not seem to offer particular advantages. However, chronic intermittent exposure was performed in only one study, and further research is needed to corroborate this hypothesis. As for direct administration of high-dose VPA during adulthood, consistent results (a social preference deficit) were found with both administration protocols. Conversely, Ilie et al. [74] observed an increase in aggressive behavior at 18 µM, whereas Li et al. [76] found decreased aggression at 500 µM. Despite these conflicting results, adult administration seems to be an effective way to shape the zebrafish ASD model, as well as a possibly time-saving and more practical approach. However, further analysis is needed to confirm the validity of the proposed model and define any long-term effects, desired and otherwise. Taken together, these findings support the hypothesis that social behavior is strictly dependent on the timing and duration of VPA exposure and on the concentration used.

Furthermore, it is worth pointing out that prenatal exposure to high VPA dosages could negatively impact locomotor activity, both in larvae and in adult zebrafish, and it is reasonable to suggest that a significant locomotor impairment could have tangible repercussions on behavioral test performances in zebrafish, whether it is the anxiety or the social phenotype that is being investigated. Indeed, as already outlined, there is a substantial difference between locomotion and exploration. Only high VPA dosages seem to negatively affect locomotion, causing significant and persistent locomotor dysfunction. Liu et al. [60] observed a persistent, significant locomotor impairment at 42 dpf after acute (7-h) exposure to 100 µM VPA. Conversely, the chronic treatment (7 h/day for 6 days) carried out by the same authors using 20 µM VPA did not produce an overlapping locomotor phenotype [60]. Zimmermann et al. [42] also observed a locomotor impairment, in their case, with 48 µM VPA administered for 48 hpf. Moreover, a locomotor deficit, albeit mild, was observed after exposing larvae to 25 µM VPA from 10 to 24 hpf [64]. In contrast, some authors evaluating fish at between 5 dpf and 180 dpf did not observe any locomotor impairment in zebrafish that had been exposed to 5–30 µM VPA [71,73], even though this range seems to allow for the detection of social impairment [78]. Although no severe or life-threatening locomotor impairment was detected for the VPA doses used, the use of VPA in low concentrations might be encouraged to prevent any secondary undesired influence on social behavior.

We would also like to briefly discuss the effect of VPA on visual systems. Messina et al. [79] used early exposure to 1 µM VPA to investigate behavioral and biological lateralization in zebrafish. They observed impairment of left visual bias and a reduction of the typical lateralized response to social stimuli, also evident at the molecular level. Their findings corroborate evidence that visual processing defects, be it at the proximal or the distal level of the central visual system, can affect social behaviors in models of neurodevelopmental disorders, such as ASD [125]. Furthermore, with regard to the impact of sight on behavior, DeOliveira-Mello et al. [64] and Bailey et al. [71] suggested that a VPA-induced visual impairment may contribute to impairments on behavioral tests (social, behavioral ones especially) in zebrafish. Even though DeOliveira et al. [64] found that exposure to 25 µM VPA did not produce a detectable and persistent visual impairment, only a transient delay in maturation of the optic nerves and retinal cells, with subsequent normalization of those parameters at 5 dpf, they correctly hypothesized that higher dosages could lead to a more protracted impairment or to incomplete visual recovery. On the other hand, Baronio et al. [73] reported that this same dosage was sufficient to induce a persistent ASD-like social behavior in adult zebrafish, further supporting the advantage and convenience of using lower dosages of VPA to obtain a valuable model of social impairment while preventing visual impairment. Notably, most protocols led to a viable phenotype, but the persistence of behavioral impairments and the occurrence of side effects varied considerably depending on the specific conditions applied.

While the present manuscript aimed to review the scientific literature on zebrafish aged more than 7 dpf, we wish to mention the conflicting findings on sleep-like behavior at 6 dpf since sleep disorders seem to particularly affect children with ASD [126]. Using the same L/D activity test adopted by Robea et al. [63], but in contrast with their findings, DeOliveira-Mello et al. [64] found an alteration of the sleep pattern in VPA-treated zebrafish. These findings could be explained by a reduction in the number of serotoninergic cells within the pineal gland, thus leading to a dysfunction in melatonin production and, consequently, to an alteration in circadian cycle regulation [127]. Considering that serotoninergic deficiency can also explain aggressive behavior [128], further studies may help to better elucidate the pathogenesis of ASD-like and ASD-associated symptoms.

Finally, this review shows that no single and well-standardized VPA-induced autism-like model has yet been established in zebrafish. It clearly emerged that larval/adult zebrafish exposure to very different dosages of VPA can determine prolonged and generally persistent autism-like traits at both morpho-structural and behavioral levels. Social behavior impairments emerge prominently, although in this area, too, the data are not always consistent: in some protocols, very low doses of VPA induced long-term impairments, while in contrast, some research groups using higher VPA doses observed only transient behavioral changes. In general, concentrations lower than 10 µM seem to lead to conflicting long-term findings, whereas those higher than 25 µM seem to lead to unintended side effects, such as locomotor impairment and visual system disruption, which can confound the data.

## 5. Conclusions

Albeit, on the basis of a search carried out in only two databases, PubMed and Scopus, we found that no ideal VPA-induced zebrafish model of autism has yet been established. The results of the literature review showed that 48 µM VPA, mainly administered at 48 hpf, appears to be the most commonly applied protocol (*n* = 5). However, early exposure to 10–25 µM VPA for between ≃24 and 120 hpf seems to be enough to obtain a solid behavioral phenotype in an ASD-like zebrafish model, preventing confounding side effects and reducing both mortality and suffering in accordance with the principles of replacement, refinement, and reduction (3Rs).

## Figures and Tables

**Table 1:** An overview of VPA-induced ASD-like models in adult zebrafish. HPLC = high-performance liquid chromatography; L/D = light/dark; RT-qPCR = quantitative reverse transcription polymerase chain reaction; VPA = valproate; RA = retinoic acid; h/d/mpf = hours/days/months post-fertilization; AChE = acetylcholine esterase.

Authors	Strain *	Administration—Timing and Duration	Administered Dose (µM)	Test Timing	Molecular Analysis **	Social Behavioral Analysis **	Non-Social Behavioral Analysis **	Non-Strictly Behavioral Analysis **	Undesired Effects
Studies conducted in juvenile specimens (younger than 90 dpf) (*n* = 8)									
Liu X. et al., 2016 [60]	AB	Chronic exposure: from 24 hpf for 7 h/day for 6 days OR Acute exposure: from 24 hpf for 7 h	20 (chronic), 100 (acute)	30 dpf	/	Social preference	L/D response	/	/
Wang J. et al., 2024 [61]	AB	8–120 hpf	5, 50, 500	10–13 dpf	Immunostaining	Shoaling, social contact, mirror biting, shoaling L/D background exploration	L/D response	Morphology, startle response	/
Dwivedi S. et al., 2019 [62]	AB	4–120 hpf	75	7 dpf, 21 dpf	qRT-PCR, Western blot (7 dpf)	Social preference (21 dpf)	Thigmotaxis (7 dpf)	Circling behavior, inattentive behavior test (7 dpf)	/
Robea M.A. et al., 2021 [63]	AB	24 hpf, 48 hpf, 72 hpf	48	6 dpf, 42 dpf	/	Social preference, mirror biting (42 dpf)	Hyperactivity, L/D activity (sleep analysis (6 dpf)	/	/
DeOliveira-Mello L. et al., 2023 [64]	Turku	10–24 hpf	25	≃7 dpf, 30 dpf	Serotoninergic immunostaining, qRT-PCR (≃7 dpf)	/	L/D activity (sleep-like behavior) (≃7 dpf)	Optomotor behavior, color preference (30 dpf), length measurement of optic nerve zones (≃7 dpf)	Delay in visual system development (no visual impairment in adult age)
Geng Y. et al., 2023 [65]	EKW	0–72 hpf	1	21 dpf	/	Social contact (ZeChat)	/	/	/
Karimi Z. et al., 2023 [66]	AB	4–120 hpf	1	7 dpf, 21 dpf, 42 dpf	qRT-PCR (7 dpf)	Social preference (21, 42 dpf)	Thigmotaxis (7 dpf), inattentive behavior test (7 dpf)	/	/
Rahmati-Holasoo H. et al., 2023 [67]	AB	0–48 hpf	48	10–13 dpf	qRT-PCR	Shoaling, social contact, mirror biting, shoaling L/D background exploration	/	/	/
Studies conducted in adult specimens (90 dpf and older) (*n* = 9)									
Blazina A.R. et al., 2013 [68]	AB/TU	10 min	88, 265, 884	240 dpf	/	Shoaling L/D background exploration	Bottom dwelling	Motor coordination	Altered motor coordination for very high-dose VPA
Lee Y. et al., 2013 [69]	n.s.	3 h/day at 120 hpf	2000	5–15 dpf, 95 dpf	qRT-PCR, immunostaining (5–15 dpf)	/	Learning (passive avoidance), bottom dwelling (95 dpf)	/	/
Dozawa M. et al., 2014 [70]	RW, gfap:GFP, elavl3:GFP	10–48 hpf, 2 or 7 days at 150–180 dpf	1000, 1500, 2000 (embryo), 500 (adult)	150–180 dpf	Cell lineage analysis, immunostaining, ISH, Western blot, qRT-PCR	/	/	/	/
Bailey J.M. et al., 2016 [71]	AB	4–120 hpf	0.5, 5, 50	Adult	/	Shoaling	Hyperactivity, bottom dwelling, predator avoidance, novel tank	Startle reflex habituation	Possible visual or motor impairment for higher doses of VPA or RA
Zimmermann F.F. et al., 2017 [72]	AB	0–48 hpf	48	120 dpf	qRT-PCR, HPLC, enzymatic activity assays	/	/	/	/
Baronio D. et al., 2018 [73]	Turku	10–24 hpf	25	5 dpf, 180 dpf	HPLC, qRT-PCR	Social preference (180 dpf)	L/D response (5 dpf), hyperactivity (180 dpf)	/	/
Ilie O.-D. et al., 2022 [74]	AB	For 2 days and for 11 days at 180–240 dpf	18	180–240 dpf	/	Social preference, mirror biting	/	/	/
Velázquez-Landa X. et al., 2023 [75]	AB	0–48 hpf	48	180 dpf	/	Sexual behavior recording	/	/	/
Li X. et al., 2024 [76]	AB	4 days	500	Adult	qRT-PCR, body cortisol extraction	Social preference, mirror biting	Bottom dwelling, hyperactivity	/	/
Studies conducted in both juvenile and adult specimens (*n* = 4)									
Zimmermann F.F. et al., 2015 [42]	n.s.	0–48 hpf	48	6, 30, 70, 120 dpf	/	Social preference, mirror biting (70 and 120 dpf)	Hyperactivity, bottom dwelling (6, 30, 70, 120 dpf)	/	/
Liu C.X. et al., 2016 [77]	TU	24–48 hpf	500, 1000	1, 3, 7, 10, 13, 15, 30, 60 dpf	qRT-PCR	/	/	/	/
Joseph T.P. et al., 2022 [78]	AB	8–108 hpf	5, 10, 20, 30, 40	5–7, 13, 90–120 dpf	AChE activity, Western blot (90–120 dpf)	Social contact (13 dpf) social preference (90–120 dpf)	Hyperactivity, bottom dwelling (90–120 dpf) L/D response (5–7 dpf)	/	/
Messina A. et al., 2024 [79]	AB	5–29 hpf, 5–53 hpf	1	21, 28, 90 dpf	qRT-PCR (90 dpf)	Visual mirror (21 dpf) social preference (28 dpf)	/	/	/

* wild-type strain; n.s. not specified; TU = Tübingen; RW = RIKEN Wako; EKW = Ekkwill. ** If test timing is not specified, see column “Test timing”.

**Table 2:** An overview of the main objective(s) of the reviewed studies.

Authors	Objective(s)	Key Findings
Studies conducted in juvenile specimens (younger than 90 dpf) (*n* = 8)		
Liu X.et al., 2016 [60]	Evaluation of VPA effects on simple and complex behaviors of juvenile zebrafish with different exposure procedures.	Impairment of social preference by chronic exposure to VPA 20 µM, while no effect on other simple behaviors (locomotor activity/anxiety/behavioral responses to light change). Impairment of locomotor activity by acute exposure to VPA 100 µM, but no effect on other behaviors.
Wang J. et al., 2024 [61]	Validation of VPA-induced ASD-like condition in zebrafish as an alternative model for investigating the mechanisms underlying ASD.	Induction of autism-like behavior and social behavior impairment in zebrafish larvae exposed to either VPA 25 or 50 μM. Vitamin A-induced amelioration of VPA-induced social impairment and neurotoxicity, through oxidative damage and apoptosis attenuation.
Dwivedi S. et al., 2019 [62]	Development of a cost- and time-effective zebrafish model with quantifiable parameters to facilitate mechanistic studies and high-throughput screening of new molecules for ASD.	Non-social behavioral impairment, and neurodevelopmental-related gene dysregulation induced by VPA 75 μM, assessed at 7 dpf. Social behavioral impairment assessed at 21 dpf. Effect of positive and negative control drugs on VPA induced behavioral despair.
Robea M.A. et al., 2021 [63]	Replication of findings on the impact of VPA on social behavior in zebrafish, and extension of the same by adding sleep observations.	Hyperactivity induced in VPA 48 μM exposed zebrafish larvae for 24 h. Social behavioral impairment induced by VPA 48 μM exposed zebrafish larvae for 24–48–72 h. No significant effects on sleep nor aggression in VPA 48 μM exposed zebrafish larvae for 24–48–72 h.
DeOliveira-Mello L. et al., 2023 [64]	Investigation, through the VPA-induced zebrafish model, of the potential visual processing impairment in ASD phenotype.	Larval retinal abnormalities after embryonic exposure to VPA 25 μM for 24 h, with normalization at 5 dpf and no visual impairment. Abnormal sleep-like behavior at assessed at 7 dpf after VPA 25 μM exposure for 24 h.
Geng Y. et al., 2023 [65]	Development of an unsupervised machine learning-based social behavioral analysis to assess the effects of neuroactive chemicals on social behavior.	Impairment of social behavior, assessed at 21 dpf in VPA 1 μM exposed larvae for 72 hpf. Rescue of social impairment by D3-agonist neuroactive compounds acutely administered for 1 h before social preference test at 21 dpf.
Karimi Z. et al., 2023 [66]	Assessment of the lowest dose of VPA able to induce ASD-like behavioral phenotypes in the zebrafish model.	Significant reduction in survival rate in 5–15–25–48–75 μM exposed zebrafish larvae for 120 h. Induction of ASD-like phenotype and impairment in social interaction persisting at 42 dpf in zebrafish larvae exposed to VPA 1 μM for 120 h.
Rahmati-Holasoo H. et al., 2023 [67]	Investigation of the impact of early administration of oxytocin on ASD zebrafish model, both at behavioral and molecular level.	Behavioral and molecular improvement in VPA 48 μM exposed zebrafish larvae for 48 h, by the administration of oxytocin 50 μM both for 24 and 48 h.
Studies conducted in adult specimens (90 dpf and older) (*n* = 9)		
Blazina A.R. et al., 2013 [68]	Evaluation of zebrafish swimming behavior against a water current using the newly developed spinning task.	Reduction in swimming time against water current in the spinning task in 240 dpf adult zebrafish exposed to VPA 884 μM for 10 min. No difference in total distance traveled for VPA 88–265–884 μM exposed zebrafish larvae.
Lee Y. et al., 2013 [69]	Evaluation of the effects of acute treatment with VPA on cell proliferation in the telencephalic area of zebrafish larvae.	Transient decrease in cell proliferation in the telencephalon of VPA 2000 μM treated zebrafish larvae at 5 dpf for 3 h, with restoration of cell proliferation rate 10 days after VPA treatment. No development of severe deficits in bottom dwelling or passive avoidance responses in 95 dpf adult fish after VPA treatment.
Dozawa M. et al., 2014 [70]	Investigation of the role of VPA in the regulation of cell proliferation in the adult zebrafish optic tectum.	Inhibition of HDAC activity and upregulation of Notch signaling, with consequent reduction of cell proliferation in the optic tectum of adult zebrafish exposed to VPA 500 µM for 48 or 168 h.
Bailey J.M. et al., 2016 [71]	Determination of the impact of non-teratogenic doses of VPA on long-term behavioral effects in zebrafish.	Reduction in the dark phase of larval activity in VPA 30–50 μM exposed larvae for 120 h, but hyperactivity in VPA 15 μM exposed larvae for 120 h. Decreased shoaling behavior in VPA 5 μM treated fish.
Zimmermann F.F. et al., 2017 [72]	Investigation of purinergic signaling in the VPA-induced adult ASD-like zebrafish model.	Alteration of biochemical and molecular parameters related to purinergic system in 120 dpf adult zebrafish exposed to 48 μM during the first 48 hpf.
Baronio D. et al., 2018 [73]	Elucidation on the role of histaminergic system in VPA-induced ASD-like zebrafish model.	High mortality/malformation rate in zebrafish embryos exposed to VPA 50–35 μM for 120 h, and identification of the optimal dosage as VPA 25 μM for 120 h. Molecular and neurochemical changes in histaminergic, noradrenergic, and dopaminergic systems induced by VPA 25 μM treatment for 120 h.
Ilie O.-D. et al., 2022 [74]	Investigation of rotenone influences social and aggressive behavior in zebrafish.	Social and aggressive behavior disruption in VPA 18 µM acutely (2-day-long) and chronically (11-day-long) exposed adult zebrafish (180–240 dpf).
Velázquez-Landa X. et al., 2023 [75]	Investigation of sexual behavior in ASD, using a VPA-induced zebrafish model.	Alteration of sexual behaviors and oviposition in adult zebrafish (180 dpf) exposed in the first 48 h of life to VPA 48 µM.
Li X. et al., 2024 [76]	Investigation of gene transcription changes, social behavior, aggression, anxiety, and locomotion in adult VPA-induced zebrafish model.	Social and aggressive behavior deficit in adult zebrafish exposed to VPA 500 µM for days. Increased anxiety in adult zebrafish exposed to VPA 500 µM for days.
Studies conducted in juvenile and adult specimens (*n* = 4)		
Zimmermann F.F. et al., 2015 [42]	Performance of behavioral screening and establishment of social interaction deficit in VPA-induced zebrafish model of ASD.	Increase in hyperactive behavior at 6 dpf (but not at 30–70–120 dpf) zebrafish exposed to VPA 48 µM for 48 hpf. Increase in anxiety up to 70 dpf. Decrease in anxiety-related behavior at 120 dpf. Social behavior impairment at 70 and 120 dpf. No difference in aggressive behavior.
Liu C.X. et al., 2016 [77]	Investigation of temporal, spatial, and isoform-specific expression patterns and transcript expression effects of *shank3* in VPA-induced zebrafish model of ASD.	Altered differential expression of shank3 isoforms at different time points (1 to 60 dpf) in zebrafish embryos exposed to 500 and 1000 µM VPA at 24–48 hpf.
Joseph T.P. et al., 2022 [78]	Assessment of the neuroprotective function of duloxetine in VPA-induced zebrafish model of ASD.	Attenuation hyperactivity, anxiety-like behavior, and social deficit by duloxetine 20 µM, in juvenile zebrafish (5–7,13,90,120 dpf) treated with VPA 10 µM from 8 to 108 hpf. Persistence up to 120 dpf of hyperactivity, increased anxiety-like behavior, and social deficits in VPA-only treated group.
Messina A. et al., 2024 [79]	Investigation of brain lateralization in VPA-induced zebrafish model of ASD.	Decrease in social preference and reduction of left visual bias in 28 dpf zebrafish exposed to VPA 1 µM for 24 and 48 hpf. Neutralization of the asymmetric distribution of genes responsible for brain lateralization assessed at 90 dpf in zebrafish exposed to VPA 1 µM for 24 and 48 hpf.

VPA = valproate; ASD = autism spectrum disorder; h/d/mpf = hours/days/months post-fertilization.

## Data Availability

The original contributions presented in this study are included in the article. Further inquiries can be directed to the corresponding author(s).
